# Gefitinib (‘Iressa’, ZD1839) and new epidermal growth factor receptor inhibitors

**DOI:** 10.1038/sj.bjc.6601550

**Published:** 2004-02-03

**Authors:** G Blackledge, S Averbuch

**Affiliations:** 1 AstraZeneca, Mereside, Alderley Park, Macclesfield, Cheshire SK10 4TG, UK; 2 AstraZeneca, OW3-236, 1800 Concord Pike, PO Box 15437, Wilmington, DE 19850-5437, USA

**Keywords:** EGFR-targeting agents, gefitinib, erlotinib, cetuximab

## Abstract

The epidermal growth factor receptor (EGFR) is a promising target for cancer therapy and a number of EGFR-targeted agents have been developed. Those most advanced in development are the EGFR tyrosine kinase inhibitors gefitinib (‘Iressa’, ZD1839) and erlotinib (‘Tarceva’, OSI-774), and the monoclonal antibody cetuximab (‘Erbitux’, IMC-C225). This review provides a clinical overview of these agents, highlighting their antitumour activities in different tumour types. Epidermal growth factor receptor–targeted agents are generally well tolerated and are not typically associated with the severe adverse events often seen with cytotoxic chemotherapy. Gefitinib is the agent with the most extensive clinical experience, particularly in non-small-cell lung cancer (NSCLC). Recently, gefitinib became the first-approved EGFR-targeted agent, for use in patients with previously treated advanced NSCLC in Japan, the USA and other countries. Further studies are required to explore the full potential of these novel agents either as monotherapy or combination therapy.

Conventional cytotoxic anticancer agents have limited efficacy and a narrow therapeutic index. Identification of molecular targets important for cancer cell proliferation and survival has provided an opportunity for improved efficacy and more selective action; on tumour rather than normal tissue (Baselga, 2002; Herbst and Kies, 2002).

One such target is the epidermal growth factor receptor (EGFR), which is highly expressed in a variety of solid tumours. Expression correlates with disease progression, poor survival, poor response to therapy and resistance to cytotoxic agents (Arteaga, 2002). The EGFR is a transmembrane glycoprotein comprising an extracellular ligand-binding domain, a transmembrane hydrophobic domain and an intracellular domain with tyrosine kinase activity involved in signal transduction (Figure 1

**Figure 1:**
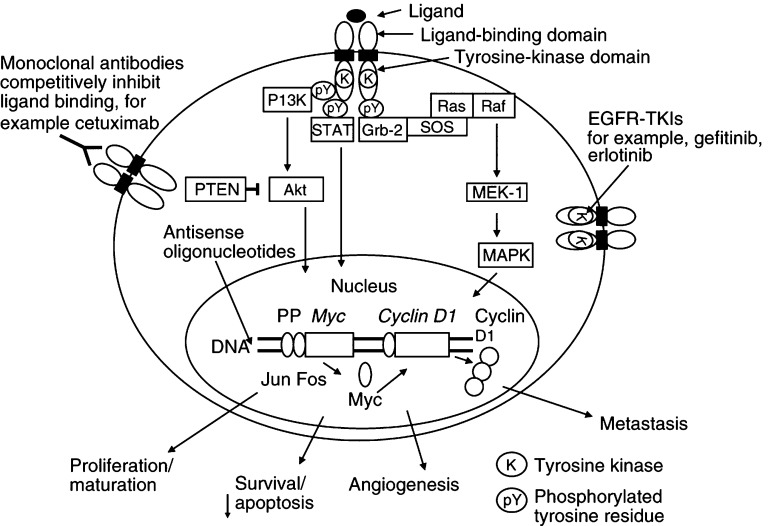
EGFR signalling and anti-EGFR approaches (reproduced with permission from: Baselga (2002); ©AlphaMed Press 1083–7159).

). Upon ligand binding, receptor dimerisation activates tyrosine kinase activity and tyrosine autophosphorylation. This initiates a signalling cascade that leads to cell proliferation, increased angiogenesis, invasion and metastasis and decreased apoptosis (Baselga, 2002).

A variety of strategies to target EGFR signalling have been investigated, including: (1) small molecule tyrosine kinase inhibitors that prevent ATP from binding to the intracellular tyrosine kinase domain of EGFR, thereby inhibiting tyrosine kinase activity and autophosphorylation, and subsequent signal transduction; (2) monoclonal antibodies that target the extracellular ligand-binding domain or bispecific antibodies that also target epitopes on the surface of immune effector cells; (3) immunotoxin conjugates using cytotoxic single-chain fragment variable antibodies conjugated to toxins such as pseudomonas endotoxin A; (4) EGF vaccines such as EGF-P64k that contained recombinant human EGF conjugated to P64k, a highly immunogenic recombinant bacterial protein; (5) antisense oligonucleotides to block the translation of the ligand or the EGFR (Figure 1) (Baselga, 2002). The first two of these approaches have proved to be the most successful (Table 1

**Table 1:** EGFR-targeted therapies in clinical development

**Class of agent**	**Agent**	**Phase of development**
*EGFR tyrosine kinase inhibitors*		
	Gefitinib, ZD1839	III/marketed
	Erlotinib, OSI-74	III
	Canertinib, CI-1033	II
	EKB-569	II
	Lapatinib, GW572016	II
*Monoclonal antibodies*		
	Human–murine chimeric monoclonal antibodies	
	Cetuximab, IMC-C225	III/pre-registration
	Fully humanised monoclonal antibodies	
	ABX-EGF	II
	EMD-72000	II
	Thera CIM-h-R3	II
	HuMax-EGFR	I/II

).

This review will concentrate on the agents most advanced in clinical development: the EGFR tyrosine kinase inhibitors gefitinib (‘Iressa’, ZD1839) and erlotinib (‘Tarceva’, OSI-774), and the chimeric human–mouse monoclonal antibody cetuximab (‘Erbitux’, IMC-C225) (Table 2

**Table 2:** Clinical development of gefitinib, erlotinib and cetuximab

**Agent**	**Class of agent**	**Phase I trial design**	**MTD**	**Dose selection parameters**	**Dose selected for further study**	**Tumour types**	**Phase of development**
Gefitinib	EGFR-TKI	Monotherapy up to 1000 mg day^−1^	⩾700 mg day^−1^	Optimum biological dose: tumour response/stable disease, target activity and pharmacokinetics	250 and 500 mg day^−1^	NSCLC, SCCHN, colorectal, breast	III/marketed (NSCLC)
Erlotinib	EGFR-TKI	Three monotherapy regimens (100–1600 mg) administered: once weekly every 3 out of 4 weeks; 3 consecutive days for 3 weeks; daily for 3 weeks	150 mg day^−1^	MTD	150 mg day^−1^	NSCLC, SCCHN, pancreatic	III (NSCLC, pancreatic)
Cetuximab	Monoclonal antibody	Administered intravenously as single infusion (5–100 mg m^−2^); weekly for 4 weeks (5–100 mg m^−2^); weekly for 4 weeks combined with cisplatin (400 mg/m^2^)	Saturates systemic clearance, 200–400 mg m^−2^	Optimum biological dose: pharmacokinetics, EGFR saturation	Loading dose 400 mg m^−2^ in week 1 followed by weekly maintenance dose of 250 mg m^−2^	SCCHN, colorectal, NSCLC	III/preregistration, (SCCHN, colorectal)

MTD=maximum tolerated dose; NSCLC=non-small-cell lung cancer; SCCHN=squamous-cell carcinoma of the head and neck.

). Gefitinib has recently received approval, the first for an EGFR-targeted agent, for the treatment of patients with previously treated advanced non-small-cell lung cancer (NSCLC) in Japan, the USA and other countries. Erlotinib is currently in the follow-up stage of three large Phase III trials and cetuximab is at the pre-registration stage for colorectal cancer.

## PRECLINICAL DATA

As monotherapy, gefitinib, erlotinib and cetuximab were antiproliferative and increased apoptosis in different cancer cell lines and human tumours xenografted to immunodeficient mice (Ciardiello *et al*, 2000; Ciardiello and Tortora, 2001). Interestingly, although there was a spectrum of dose-dependent antitumour activity ranging from dramatic tumour regression to *de novo* resistance when gefitinib was tested in multiple human tumour xenograft studies, the level of EGFR expression did not predict tumour response (Wakeling *et al*, 2002). Many studies demonstrated additive or synergistic antitumour effects when combining EGFR-targeted agents with chemotherapy, ionising radiation or other novel agents (Milas *et al*, 2000; Sirotnak *et al*, 2000; Tortora *et al*, 2001; Bianco *et al*, 2002; Buchsbaum *et al*, 2002; Huang *et al*, 2002; Magne *et al*, 2002; Normanno *et al*, 2002; Williams *et al*, 2002; Prewett *et al*, 2003; Solomon *et al*, 2003; Xu *et al*, 2003).

## CLINICAL OVERVIEW

Epidermal growth factor receptor-targeted agents have been investigated in several human cancers. Many studies have involved patients with tumours that widely express EGFR, including NSCLC, colorectal cancer, squamous-cell carcinoma of the head and neck (SCCHN) and breast cancer. Existing treatments improve survival times, but many patients with advanced disease relapse and require further therapy to palliate symptoms and improve survival. This highlights the significant unmet need for novel, targeted agents (Herbst and Kies, 2002; O’Dwyer and Benson, 2002; Herbst and Langer, 2002; Cohen, 2003; Cohen *et al*, 2003a).

### Gefitinib

Four multicentre, open-label, Phase I trials investigated the tolerability and efficacy of oral gefitinib (up to 1000 mg day^−1^) in patients with a variety of solid tumours, including NSCLC (Baselga *et al*, 2002; Herbst *et al*, 2002; Ranson *et al*, 2002; Nakagawa *et al*, 2003). As gefitinib is not a traditional cytotoxic agent, dose selection for further study was based on identification of the optimum biological dose, combining maximum efficacy with minimum adverse events (AEs).

Gefitinib was generally well tolerated; the most common AEs were mild/moderate (grade 1/2) reversible rash and diarrhoea, whose incidence and severity increased with increasing dose. Gefitinib was not typically associated with the cytotoxic AEs of chemotherapy. The maximum tolerated dose (MTD) was ⩾700 mg day^−1^. Promising antitumour activity was observed in a number of tumour types, particularly NSCLC; 10 out of 100 NSCLC patients experienced a partial tumour response (Herbst *et al*, 2002; Ranson *et al*, 2002; Nakagawa *et al*, 2003). Partial responses and disease stabilisation were observed at doses ⩾150 mg day^−1^ with no suggestion that higher doses provided greater antitumour activity (Herbst and Kies, 2002; Ranson *et al*, 2002). Similarly, pre- and post-treatment skin biopsy results from cancer patients revealed that EGFR signalling was inhibited at doses ⩾150 mg day^−1^, with no clear dose dependence above this level (Albanell *et al*, 2002). Based on these results, two doses below the MTD were chosen for evaluation in Phase II studies: 250 mg day^−1^, at which dose responses had been seen with minimum toxicity, and 500 mg day^−1^, the highest dose that has been tolerated on prolonged treatment.

#### Gefitinib monotherapy in NSCLC

Two large-scale Phase II, multicentre, dose-randomised, double-blind, parallel-group studies, Iressa Dose Evaluation in Advanced Lung Cancer (IDEAL) 1 and 2, evaluated gefitinib monotherapy (250 and 500 mg day^−1^) in patients with locally advanced or metastatic NSCLC who had received platinum-based chemotherapy (Fukuoka *et al*, 2003; Kris *et al*, 2003). Doses were given daily until disease progression or withdrawal due to intolerable toxicity. Patients in IDEAL 1 were recruited from Europe, Japan, Australia and South Africa (103 patients at 250 mg day^−1^; 106 patients at 500 mg day^−1^) and must have received one or two prior chemotherapy regimens, at least one of which contained platinum. Patients in IDEAL 2 were recruited from the USA (102 patients at 250 mg day^−1^; 114 patients at 500 mg day^−1^), and must have received at least two prior regimens including platinum and docetaxel given either concurrently or separately. Disease-related symptoms were measured weekly using the independently validated Lung Cancer Subscale (LCS) of the Functional Assessment of Cancer Therapy-Lung (FACT-L) quality-of-life questionnaire (Cella *et al*, 2002). Using the LCS, the severities of seven disease-related symptoms are recorded on a five-point Likert scale where the maximum (best) score possible is 28. All patients in IDEAL 2 were symptomatic, with a baseline LCS score of ⩽24 points.

**Figure 2:**
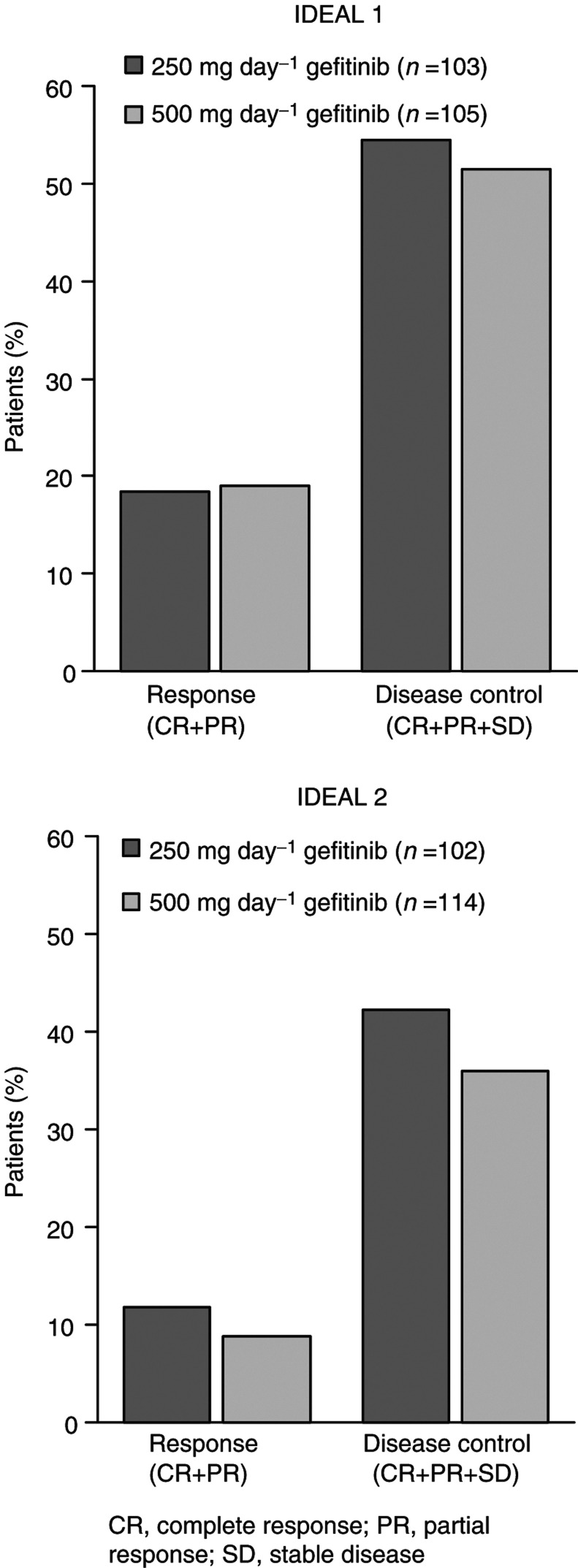
Gefitinib antitumour activity in advanced NSCLC: IDEAL 1 and 2.

Both doses showed clinically meaningful antitumour activity with no greater efficacy at the higher dose (Figure 2

). Adverse events were fewer and less severe at the lower dose. Accordingly, 250 mg day^−1^ gefitinib (approximately one-third of the MTD) is the recommended/optimum biological dose in NSCLC, causing minimum AEs without compromising efficacy. Only results for this dose will be discussed here. For IDEAL 1 and 2, respectively, response rates were 18.4 and 11.8%, disease control rates were 54.4 and 42.2%, median progression-free survival was 2.7 and 1.9 months and median overall survival was 7.6 and 6.5 months. Objective responses were achieved regardless of the number of prior chemotherapy regimens.

Many patients’ disease-related symptoms improved significantly, important because many NSCLC patients have frequent and severe symptoms. Symptom improvement rates for IDEAL 1 and 2 were 40.3 and 43.1%, with a median time to improvement of 8 and 10 days, respectively. Symptom improvement correlated with objective tumour response and was associated with increased overall and progression-free survival (Herbst and Kies, 2002). In IDEAL 2, the median overall survival for patients with symptom improvement was 13.6 months, compared with 3.7 months for patients without symptom improvement (Douillard *et al*, 2002).

In IDEAL 1 and 2, 24 and 34% of patients had an improved quality of life, with a median time to improvement of 29 and 30 days, respectively. Most patients with a partial/complete response or stable disease also showed improved quality of life (Natale and Zaretsky, 2002).

The most common AEs were skin rash (47 and 43% in IDEAL 1 and 2, respectively) and diarrhoea (40 and 48%, respectively). The majority of these AEs were Common Toxicity Criteria grade 1 and, across both IDEAL 1 and 2, grade 3 diarrhoea and skin rash were each observed in only one patient. The incidences of dose reductions (<1%) and withdrawals (1.9 and 1.0% in IDEAL 1 and 2, respectively) due to AEs were also low (Schiller *et al*, 2002).

#### Gefitinib combination therapy in NSCLC

Two Phase I trials using carboplatin/paclitaxel and cisplatin/gemcitabine, respectively, showed combination therapy to have acceptable tolerability and no unexpected or cumulative toxicity (Miller *et al*, 2001; Gonzalez-Larriba *et al*, 2002).

Two multinational, randomised, double-blind, placebo-controlled Phase III studies investigated gefitinib combined with standard platinum-based first-line chemotherapy regimens. The ‘Iressa’ NSCLC Trials Assessing Combination Treatment (INTACT) 1 and 2 involved more than 2100 chemonaive patients with advanced NSCLC (Giaccone *et al*, 2002; Johnson *et al*, 2002). Disappointingly, there were no improvements in overall survival or other efficacy outcomes in the first-line setting. However, these placebo-controlled studies confirmed the favourable safety profile of gefitinib observed in the IDEAL trials. The combination toxicity profile was similar to that of chemotherapy alone except for the addition of dose-dependent diarrhoea and skin rash.

#### Gefitinib therapy against other cancers

A Phase II study investigating gefitinib monotherapy (500 mg day^−1^) in patients with recurrent or metastatic SCCHN showed promising antitumour activity and acceptable toxicity. Of 47 evaluable patients, the response rate was 10.6% and the disease control rate 53%. The median time to disease progression and overall survival were 3.4 and 8.1 months, respectively. This compares with median survival times of 6–8 months from Phase III trials of other agents in this setting. Skin toxicities, never greater than grade 2, were observed in 48% of patients, and diarrhoea was observed in 50% of patients (at grade 3 in three patients) (Cohen *et al*, 2003a). A Phase II trial is recruiting patients (*n*=63) with recurrent SCCHN to investigate gefitinib monotherapy (250 mg day^−1^). Of the 14 patients currently evaluable for response, four had stable disease. Toxicity was assessed in 17 patients; the most common AEs were grade 1/2 rash in 50% of patients and grade 1/2 diarrhoea in 20% of patients, with no grade 3/4 AEs (Cohen *et al*, 2003b). Further data from this patient population will be needed to determine whether 250 or 500 mg day^−1^ has optimum activity in recurrent SCCHN.

A Phase II study investigating combination therapy of gefitinib (500 mg day^−1^) with FOLFOX-4, a triple-combination standard treatment regimen for patients with advanced colorectal cancer, showed antitumour activity. For untreated patients, the response rate was 75% and for patients who had relapsed after chemotherapy, 29%. These values compare with historical response rates of 30–55 and 9%, respectively. Combination therapy was generally well tolerated with the most common grade 3/4 AE being diarrhoea (Cho *et al*, 2003).

Two Phase II studies investigated gefitinib monotherapy (500 mg day^−1^) in patients with advanced breast cancer. In the first study (*n*=31), 10 patients (32%) had stable disease for ⩾3 months (Baselga *et al*, 2003). The second study involved nine evaluable patients with acquired tamoxifen-resistant oestrogen-receptor-positive breast cancer and 18 evaluable patients with oestrogen-receptor-negative breast cancer. Of the tamoxifen-resistant patients, one patient had a partial response and five had stable disease. Of the oestrogen-receptor-negative patients, one patient had a partial response and one patient had stable disease (Robertson *et al*, 2003).

### Erlotinib

In Phase I trials involving pretreated patients with advanced solid tumours including NSCLC, three monotherapy regimens were administered (dose range 100–1600 mg): once weekly every 3 out of 4 weeks; on 3 consecutive days for 3 weeks; daily for 3 weeks. The MTD was not reached in the once-weekly schedule and diarrhoea was the dose-limiting toxicity in the once-daily schedule, at 200 mg day^−1^ (Siu *et al*, 1999). Common AEs were skin rashes and diarrhoea. Using conventional chemotherapy dose-selection methods, 150 mg day^−1^ (MTD) was selected for Phase II studies (Ciardiello and Tortora, 2001; Kim and Murren, 2002).

#### Erlotinib in NSCLC

In a Phase II trial, 56 patients with EGFR-positive NSCLC recurrent or progressive after platinum-based chemotherapy received erlotinib monotherapy. At 12 weeks, 10.7% had a confirmed partial response and 33.9% had stable disease. Treatment was generally well tolerated and the most common AE was a maculopapular acneiform rash in 78% of patients. No patients discontinued treatment due to toxicity and only two patients had dose reductions to 100 mg day^−1^ (Perez-Soler *et al*, 2001).

Current Phase III trials in NSCLC are investigating monotherapy in refractory patients randomised to receive erlotinib or placebo and first-line combination therapy with carboplatin/paclitaxel or gemcitabine/cisplatin (Kim and Murren, 2002). Preliminary results from the first-line combination trials have shown that combination with erlotinib did not result in an improvement in overall survival compared with chemotherapy alone.

#### Erlotinib and other cancers

Erlotinib monotherapy has antitumour activity in other tumour types. Out of 114 patients with SCCHN, 78 were evaluable for response: 12.8% had a partial response and 29.5% had disease stabilisation. The most common AE (*n*=114) was acneiform rash in 72% of patients (Senzer *et al*, 2001). Of 30 evaluable patients with pretreated advanced refractory ovarian cancer, 6.7% had a partial response and 10% had disease stabilisation at 5–6 months. Treatment was generally well tolerated and rash was the most common AE, in 88% of patients (Finkler *et al*, 2001).

A Phase III trial is investigating erlotinib (100 mg day^−1^) and gemcitabine combination therapy in 800 patients with pancreatic cancer (Kim and Murren, 2002).

### Cetuximab

In three consecutive open-label Phase I trials, 52 patients with advanced tumours expressing high levels of EGFR were administered cetuximab intravenously as a single infusion (5–100 mg m^−2^), weekly for 4 weeks (5–100 mg m^−2^) or weekly for 4 weeks (up to 400 mg m^−2^) combined with cisplatin. The MTD was not reached in any study; toxicity was minimal and unrelated to dose or number of cycles administered. Common AEs were skin toxicities, fever and chills, asthenia, transient transaminase elevations and nausea. Four patients had grade 3/4 AEs. One patient receiving monotherapy had grade 3 aseptic meningitis. When cetuximab was combined with cisplatin, one patient had diarrhoea (grade 3), one patient had an anaphylactic reaction (grade 3) and one patient had both epiglottitis (grade 3) and dyspnoea (grade 4) (Baselga *et al*, 2000).

Cetuximab displays nonlinear pharmacokinetics and, due to its long half-life, can be administered weekly (Herbst and Hong, 2002). In Phase Ib clinical trials, a loading dose of 400 mg m^−2^ at week 1 followed by a weekly maintenance dose of 250 mg m^−2^ achieved almost complete saturation of EGFR in tumour tissue. This was the recommended dose for Phase II and III clinical trials (Shin *et al*, 2001).

Replacing the constant region of the original mouse monoclonal antibody with the constant region of human IgG1 reduced immunogenicity. However, 4–6% of patients experience a serious allergic event within minutes of infusion and 2% have anaphylactic reactions (O’Dwyer and Benson, 2002; Baselga, 2002).

#### Cetuximab combination therapy

Combining cetuximab with standard anticancer treatments in patients with SCCHN, colorectal or pancreatic cancer did not increase toxicity (Needle, 2002).

A Phase I study combining different doses of cetuximab with radiotherapy showed promising activity in therapy-naive patients with advanced SCCHN. Of 15 patients, 86.7% had a complete and 13.3% a partial response (Robert *et al*, 2001). A Phase II study investigated cetuximab/cisplatin combination therapy in patients with SCCHN; 12% of 78 evaluable patients with disease refractory to cisplatin responded (Kies and Harari, 2002). A Phase III study recruited 121 patients with untreated SCCHN metastatic disease to compare cisplatin and cetuximab combination therapy with cisplatin and placebo. The response rate was higher in the combination treatment group: 23 *vs* 9%. However, there was no significant difference in time to tumour progression (4.1 and 3.4 months, respectively) or overall median survival (9.2 and 8.0 months, respectively). Toxicity data was available for 64 patients. Grade 3/4 AEs were hypersensitivity (6%), neutropenia (17%) and rash/desquamation (11%) (Burtness *et al*, 2002; Kies and Harari, 2002).

Cetuximab monotherapy *vs* cetuximab combined with irinotecan was investigated in 329 EGFR-positive, irinotecan-refractory patients with metastatic colorectal cancer. The response rate for the 218 patients who received combination therapy was 17.9% and the median time to progression was 126 days. In contrast, for monotherapy patients, these values were 9.9% and 45 days. The 65 AEs potentially related to treatment were consistent with the safety profiles of the individual agents (Cunningham *et al*, 2003).

Cetuximab combination therapy with cisplatin/vinorelbine *vs* cisplatin/vinorelbine alone is being investigated as first-line treatment in patients with EGFR-positive advanced NSCLC. Preliminary response rates for 18 and 17 patients are 50 and 29%, respectively. Only two serious AEs have been related to treatment with cetuximab (Gatzemeier *et al*, 2003).

## DISCUSSION

Of the EGFR-targeting agents discussed in this review, gefitinib has undergone the most extensive clinical evaluation. While the agents share many similarities, differences in their properties and approach to clinical development might influence their clinical profile.

Gefitinib and erlotinib are administered orally, once daily, so would be more suitable for an outpatient setting. In contrast, cetuximab is administered intravenously and, as it is a chimeric human–mouse monoclonal antibody, it can cause allergic reactions (Ciardiello and Tortora, 2001; Baselga, 2002). Other monoclonal antibodies in clinical development are fully humanised antibodies that do not generate human antimouse antibodies, thereby reducing the risk of inducing hypersensitivity reactions in patients and potentially prolonging their *in vivo* lifetime.

Different approaches to dose selection have been used. As gefitinib is not a cytotoxic agent, it does not need to be given at the MTD. In NSCLC patients, the 250 mg day^−1^ recommended dose (about one-third of the MTD) showed equivalent efficacy to 500 mg day^−1^, but was associated with fewer grade 3/4 AEs, dose reductions, and withdrawals. This supports Phase I trials that show flat dose–response curves for efficacy while AEs increase with dose. Cetuximab is also dosed below the MTD, whereas erlotinib has followed a conventional cytotoxic dose-selection process, with dosing at the MTD.

All three agents have shown monotherapy activity although there are less data for cetuximab. It is harder to assess the efficacy of combination treatment over the activity of individual agents. Some studies provide promising results, whereas others demonstrate no advantage. Further studies are required to assess and optimise combination treatment with these agents.

The most common AEs for these EGFR-targeting agents are rash and diarrhoea and are higher for erlotinib, which is dosed at the MTD. These agents are not associated with the typical cytotoxic AEs affecting patients treated with chemotherapy (Ciardiello and Tortora, 2001).

In Japan, interstitial lung disease (ILD) has been observed in gefitinib-treated patients with an incidence of 1.7% (Inoue *et al*, 2003). This is higher than the worldwide reported incidence of 1% in over 92 000 patients treated (up to September 2003) and 0.38% in >39 000 patients treated as part of a compassionate-use programme (Forsythe and Faulkner, 2003). The incidence might be higher in Japanese patients due to greater awareness of ILD compared with the rest of the world, differences in ILD definitions or increased genetic susceptibility. In one retrospective study of 711 Japanese patients with lung cancer who had undergone surgical resection, 7.5% had idiopathic pulmonary fibrosis, a type of ILD (Kawasaki *et al*, 2002). Interstitial lung disease is a known complication of chemotherapy and radiotherapy in patients with lung cancer (Abid *et al*, 2001) and many patients with advanced NSCLC have no further treatment options, so the benefits of gefitinib treatment outweigh the risks of ILD.

Epidermal growth factor receptor-targeted agents have also shown promise in the treatment of patients with bronchioalveolar carcinoma (BAC), which is considered to be a subtype of adenocarcinoma of the lung without pleural, stromal or vascular invasion (World Health Organization classification). In a recent presentation at the European Cancer Conference, 13 out of 52 evaluable patients (25%) with BAC had a partial response to treatment with erlotinib (Patel *et al*, 2003). Similar results have been shown for gefitinib, with response rates of 20% (first line) and 12% (pretreated) reported in patients with advanced BAC (West *et al*, 2003).

The use of targeted agents has raised the possibility of selecting the patients most likely to respond to treatment. Although some studies involving EGFR-targeted agents selected patients according to EGFR expression, there is no evidence for an association between EGFR levels and response to small-molecule EGFR-targeted agents. Hence, there are no data to support EGFR screening to select patients who would benefit from treatment (Woodburn *et al*, 2000; Arteaga, 2002). As with conventional chemotherapy, some clinical baseline characteristics were predictive of greater response rates, for example, response rates to gefitinib were higher for women and patients with adenocarcinoma (Schiller *et al*, 2003). However, responses were observed in all groups. Further studies are required to identify the molecular profiles that predict the patients most likely to benefit from treatment with these agents.

## CONCLUSIONS

As monotherapy or combination therapy, EGFR-targeted agents have demonstrated promise in treatment of several tumour types. The most extensive clinical experience has been with gefitinib (>92 000 patients) and two large Phase II studies have demonstrated clinically relevant antitumour activity in patients with previously treated advanced NSCLC.

The EGFR agents discussed have favourable AE profiles and are not typically associated with the heavy toxicity burden of chemotherapy agents, allowing long-term treatment. The maximum efficacy for targeted agents occurs below toxic doses, allowing both gefitinib and cetuximab to be administered at doses lower than the MTD to maximise the benefit : risk ratio. Gefitinib is the first EGFR agent to be approved for cancer treatment in Japan, the USA and other countries.

These novel therapies offer new treatment strategies to cancer patients with limited treatment options and further studies are underway to explore their full potential.

‘Iressa’ is a trademark of the AstraZeneca group of companies, ‘Tarceva’ is a trademark of OSI Pharmaceuticals, Inc., ‘Erbitux’ is a trademark of ImClone Systems Incorporated of New York.
